# Supplementary material for: Targeted Metabolomics of Organophosphate Pesticides and Chemical Warfare Nerve Agent Simulants Using High- and Low-Dose Exposure in Human Liver Microsomes
Source: Metabolites. 2023 Mar 29;13(4):495. doi: 10.3390/metabo13040495 (PMC10144572; doi:10.3390/metabo13040495)
Supplement: Supplementary file 1 [file metabolites-13-00495-s001.zip › metabolites-2230702-supplementary.pdf]

Supporting Information

Targeted metabolomics of organophosphate pesticides and chemical warfare nerve agent simulants using high and low-dose exposure in human liver microsomes

Garima Agarwal [1], Hunter Tichenor [2], Sarah Roo [1], Thomas R. Lane [3], Sean Ekins [3] and Craig A. McElroy [1,*]

[1] Division of Medicinal Chemistry and Pharmacognosy, College of Pharmacy, The Ohio State University, Columbus, OH 43210, USA
[2] Department of Chemistry and Biochemistry, The Ohio State University, Columbus, OH 43210, USA
[3] Collaborations Pharmaceutical Inc., Raleigh, NC 27606, USA
* Correspondence: mcelroy.31@osu.edu

Table of Contents

Instrument parameters for OPs 2
Clearance rates and decay type of OPs at low- (MS) and high-dose (NMR) regimens 4
^{31}P NMR spectra for formothion and dimethoate (5mM) in the presence of microsomes 7
^{31}P NMR spectra of malathion (5mM) in the presence of microsomes 7
Commonly observed metabolites for different OPs 8
Linear calibration curves of representative OPs for low-dose regimen 8
Decay curves for OPs at low-(MS) and high-dose (NMR) regimens 10

Instrument parameters for OPs

Table S1: Upper limit of quantification (ULOQ, nM), lower limit of quantification (LLOQ, nM), LD50 (µM), quantification ion (positive ion mode) and retention time (mins) of OPs developed on a TSQ Quantiva connected to Thermo Vanquish. (Key correlating the abbreviated OP names with the full IUPAC names can be found in the supplemental materials).

OPs	Monoisotopic mass	Quant Ion	LLOQ	ULOQ	Retention time	LD_{50}	ISTD
Methamidophos (MMP)	141.00	94.30	0.21	3542.83	1.56	6.75[a, b]	Ace-d3
Acephate	183.01	143.20	0.08	2729.71	2.36	163.88[a, b]	Ace-d3
Dimefox	154.07	110.00	0.10	1622.06	4.40	0.21[a, b]	TCP-d6
Monocrotophos (MCP)	224.00	127.00	0.03	1120.07	4.40	1.99[a, b]	TCP-d6
Tricholphon (TCP)	519.90	109.30	0.09	971.10	4.41	26.16[a, b]	TCP-d6
Dimethoate	229.00	198.92	0.14	2180.93	4.57	33.95[a, b]	PE-d10
Mevinphos (MVP)	224.04	127.30	0.13	2230.65	4.67	0.50[a, b]	PE-d10
Schradan	286.13	135.20	0.03	524.01	4.76	0.56[a, b]	PE-d10
Phosfolan	255.02	139.92	0.45	7833.92	4.78	1.11[a, b]	PE-d10
Formothion	256.99	124.92	1.77	3886.51	4.98	30.86[a, b]	PE-d10
Mephosfolan (MPF)	269.03	196.00	0.06	928.33	5.05	31.85[a, b]	PE-d10
Phosphamidon-I (PPM-I)	299.07	127.00	0.05	1668.39	5.06	0.74[a, b]	PE-d10
Dichlorvos (DCV)	219.95	108.92	202.73	27151.78	5.08	11.50[a, b]	PE-d10
Phosphamidon-II (PPM-II)	299.07	127.00	0.05	1668.39	5.14	0.74[a, b]	PE-d10
Diisopropyl fluorophosphate (DFP)	184.07	100.92	38.56	21721.42	5.24	1.04[a, b]	PE-d10
Paraoxon-methyl (PM)	247.02	202.07	0.12	2023.15	5.33	0.26[a, b]	PE-d10
Dibrom	377.78	127.00	0.59	2626.19	5.60	6.92[a, b]	PE-d10
EMP (VX simulant)	307.06	280.40	0.15	2441.09	5.69	0.38[c, d]	PE-d10
Heptenophos	250.02	109.00	0.46	3990.26	5.74	12.17[a, b]	PE-d10
NEDPA (Tabun simulant)	274.07	247.00	0.11	1823.40	5.96	27.52[e, f]	PE-d10
Paraoxon-ethyl (PE)	275.06	219.92	0.07	545.08	6.02	0.35[a, b]	PE-d10
Methidathion (MDT)	301.96	145.20	0.10	1653.82	6.08	2.63[a, b]	PE-d10
Fenthion Oxon	262.04	216.00	0.23	942.47	6.25	3.11[a, b]	PE-d10
Phosmet	317.00	160.00	0.09	1575.70	6.36	11.31[a, b]	PE-d10
Azinphos-methyl (APM)	317.01	160.00	0.09	1575.68	6.40	0.90[a, b]	PE-d10
Ethoprophos (EPP)	242.06	214.92	0.12	1031.61	6.44	5.24[a, b]	PE-d10
Malathion	330.04	127.30	0.09	3027.00	6.56	170.96[a, b]	CPy-d10
Pyridaphenthion (PPT)	340.07	189.00	0.02	1469.16	6.78	71.78[a, b]	CPy-d10
Triazophos (TAP)	313.07	162.00	0.03	797.93	6.83	6.69[a, b]	CPy-d10
Crufomate	291.08	235.90	0.10	1714.03	6.88	103.45[a, b]	CPy-d10

OPs	Monoisotopic mass	Quant Ion	LLOQ	ULOQ	Retention time	LD_{50}	ISTD
Fenamiphos (FMP)	303.11	233.92	0.10	824.10	6.91	0.63[a, b]	CPy-d10
CMP (Cyclosarin simulant)	361.11	280.00	0.33	1383.76	6.92	0.32[g, h]	CPy-d10
Tetrachlorvinphos (TCVP)	363.90	127.00	0.08	1366.31	6.96	347.20[a, b]	CPy-d10
Chlorpyrifos Oxon (CPO)	332.95	277.83	0.09	1494.77	6.99	6.27[a, b]	CPy-d10
PiMP (Soman simulant)	363.12	279.92	1.25	11004.13	7.00	0.97[c, g]	CPy-d10
Iprobenfos (IBF)	288.09	91.00	0.05	1734.06	7.06	74.91[a, b]	CPy-d10
Bensulide	397.06	355.92	0.08	628.91	7.12	21.58[a, b]	CPy-d10
Chlorfenvinphos (CFVP)	357.97	99.00	0.04	695.28	7.18	1.06[a, b]	CPy-d10
Diazinon	304.10	96.92	0.03	821.42	7.20	3.32[a, b]	CPy-d10
Fenthion	278.02	168.00	0.22	449.11	7.21	3.64[a, b]	CPy-d10
Quinalphos	298.05	163.00	0.10	838.08	7.26	7.56[a, b]	CPy-d10
Isofenphos	345.11	244.00	0.36	2895.28	7.30	2.58[a, b]	CPy-d10
Cyanofenphos (CFP)	303.05	156.92	0.98	824.27	7.43	4.50[a, b]	CPy-d10
Phosalone	366.99	182.30	0.08	1359.40	7.50	10.36[a, b]	CPy-d10
Chlorphoxim (CPh)	332.02	110.90	0.09	3005.35	7.61	238.66[a, b]	CPy-d10
Isoxathion (IXT)	313.05	285.00	0.10	1595.86	7.74	11.36[a, b]	CPy-d10
Leptophos Oxon	393.89	155.00	0.29	10101.01	7.75	9.47[a, b]	CPy-d10
Pirimiphos Ethyl (PPE)	333.13	198.00	0.03	749.87	7.79	13.34[a, b]	CPy-d10
Pyrazophos (PZP)	373.09	222.00	0.08	1339.19	7.85	12.85[a, b]	CPy-d10
Iodofenphos (IFP)	411.84	125.00	73.61	9685.23	8.04	179.21[a, b]	CPy-d10
Chlorpyrifos (CPy)	348.93	197.97	0.04	713.08	8.05	5.98[a, b]	CPy-d10
Aspon	378.09	210.83	0.31	660.68	8.33	141.45[i]	CPy-d10
Temephos	465.99	418.92	0.98	1071.81	8.46	68.09[a, b]	CPy-d10
Merphos	298.10	169.00	101.84	13400.34	8.50	73.43[a, b]	CPy-d10
Tribufos	314.10	168.92	0.20	3179.65	8.62	3.12[a, b]	CPy-d10
Leptophos	409.87	77.00	9.71	9707.32	8.88	3.32[a, b]	CPy-d10

References: (a): Kim et. al., 2021[25]; (b) Lewis et. al., 2016[26]; (c) Misik et. al., 2015[28]; (d) Amitai et. al., 2006[30]; (e) Meek et. al., 2012[23]; (f) Sivam et. al., 1984[32]; (g) Gupta et. al., 2011[29]; (h) Amitai et. al., 2007[31]; (i) Backus et. al., 1982[24].

Clearance rates and decay type of OPs at low- (MS) and high-dose (NMR) regimens

Table S2: Intrinsic clearance rates of OPs in HLMs and the percent of OP remaining after incubation at both low- (MS) and high-dose (NMR) regimens and the metabolism type, monophasic decay (OPD) or biphasic decay (BPD), and NA for OPs that were not water soluble at the high concentration. The legend for the table is given below:

	NMR	MS	
**Cl (int)**			Biphasic in both (NMR and MS)
Low	<1	<20	Biphasic in NMR
Mid	1-10	20-100	Biphasic in MS
High	>10	>100	MS: BPD but fast half life too quick to determine with the given time points
**% Remaining**			NMR: BPD but fast half life too quick to determine with the given time points
Low	<30		Span <2*Sy.x: Clearance rate negligibe: NMR
Mid	30-80		Span <2*Sy.x: Clearance rate negligibe: both
High	>80		Span <2*Sy.x: Clearance rate negligibe: MS

			Data based on MS			Data based on NMR		
**OP**	**Decay Type**	**MW**	**Cl_{int}**	**Half-life (hour)**	**% Remaining**	**Cl_{int}**	**Half-life (hour)**	**% Remaining**
Acephate	OPD	183.17	60.987	0:22:44	91.90	8.875	1:57:08	90
APM	OPD	317.32	45.150	0:30:42	5.00	6.798	2:02:20	0
Aspon-Fast	BPD	378.40	664.459	0:02:05	13.00	3.955	4:22:51	60
Aspon-Slow			7.126	3:14:29	1.62	0.509	10:01:04	5
Bensulide	OPD	397.51	9.136	2:31:43	0.45	NA		
CFP	OPD	303.30	14.923	1:32:53	20.00			
CFVP	OPD	359.57	73.904	0:18:45	0.52			
CMP	OPD	361.33	145.549	0:09:31	6.87	1.693	10:13:57	17.6
CPh	OPD	332.74	152.708	0:09:05	20.00	NA		
CPO	OPD	334.50	61.187	0:22:39	0.55	6.707	2:35:00	10.3
CPy	OPD	350.59	12.015	1:55:21	28.00	NA		
Crufomate	OPD	291.71	155.267	0:08:56	20.00			
DCV	OPD	220.98	3.027	7:37:50	31.00	1.322	13:00:40	2
DFP	OPD	184.15	3.683	6:16:18	27.00	0.684	1:20:04	40
Diazinon	OPD	304.35	28.194	0:49:10	32.00	7.759	2:13:58	30
Dibrom-Fast	BPD	380.78	461.902	0:03:00	23.00	8.193	2:01:48	75
Dibrom-Slow			123.194	0:11:15	0.38			
Dimefox	OPD	154.13	129.832	0:10:41	80.00	0.382	12:15:36	100
Dimethoate	OPD	229.26	72.125	0:19:13	95.00	0.232	11:44:01	87
EMP-Fast	BPD	307.24	83.042	0:16:41	0.62	5.589	3:05:59	50
EMP-Slow						0.297	10:15:27	0.1
EPP	OPD	242.34	36.572	0:37:54	4.00	NA		
Fenthion	OPD	278.33	53.009	0:26:09	23.00			

			Data based on MS			Data based on NMR		
OP	Decay Type	MW	Cl_{int}	Half-life (hour)	% Remaining	Cl_{int}	Half-life (hour)	% Remaining
Fethion Oxon	OPD	265.26	17.030	1:21:23	90.00			
FMP	OPD	303.36	59.767	0:23:11	50.00			
Formothion-Fast	BPD	257.30	24.555	0:56:27	0.11	22.27	0:46:41	70
Formothion-Slow			3.495	6:36:32	0.11	2.623	6:36:19	47
Heptenophos	OPD	250.61	2.901	7:57:42	60.00	0.245	22:42:02	85.8
IBF	OPD	288.34	5.831	3:57:42	79.00	98.571	0:08:26	30
IFP-Fast	BPD	413.00	118.635	0:11:41	74.31	NA		
IFP-Slow			3.019	7:39:02	0.30			
Isofenphos	OPD	345.39	61.387	0:22:35	0.80			
IXT-Fast	BPD	313.31	107.777	0:12:52	82.00			
IXT-Slow			5.281	4:22:28	15.00			
Leptophos	OPD	412.06	5.799	3:59:01	3.51			
Leptophos Oxon	OPD	396.00	13.217	1:44:52	3.36			
Malathion-Fast	BPD	330.36	2245.523	0:00:37	20.00	11.149	1:33:14	4.5
Malathion-Slow			10.092	2:17:20	1.80	NA		
MCP	OPD	223.20	11.288	2:02:47	95.00	0.002	21:05:00	89.8
MDT	OPD	302.33	18.370	1:15:27	46.50	1.716	10:05:54	55
Merphos-Fast	BPD	298.50	127.033	0:00:37	72.00	2.169	6:23:23	60
Merphos-Slow			8.876	2:17:20	1.44			
MMP	OPD	141.13	10.590	2:10:53	97.00	1.582	10:57:01	94
MPF	OPD	269.30	4.859	4:45:15	96.00	1.296	10:41:48	97
MVP-I	OPD	224.15	6.867	3:21:51	0.75	0.008	10:53:49	82
MVP-II	OPD	224.15	10.684	2:09:44	0.36	18.911	0:54:58	98
NEDPA	OPD	274.21	24.895	0:55:40	70.00	0.742	23:20:00	57
PE	OPD	275.19	25.355	0:54:40	68.00	0.035	13:16:23	73
Phosalone	OPD	367.81	23.415	0:59:12	2.00	NA		
Phosfolan	OPD	255.30	70.545	0:19:39	95.00	1.394	12:25:38	90
Phosmet	OPD	317.32	13.607	1:41:52	4.00	NA		
PiMP	OPD	363.50	55.888	0:24:48	50.00	1.573	11:00:46	65
PM	OPD	247.14	30.354	0:45:40	68.00	0.072	2:11:23	48
PPE-Fast	BPD	333.39	45.010	0:30:48	60.00	NA		
PPE-Slow			7.072	3:15:58	3.50			
PPM-I	OPD	299.69	35.572	0:38:58	89.00	0.36	16:49:19	80
PPM-II	OPD	299.69	4.335	5:19:43	22.00	0.026	7:09:23	60
PPT	OPD	340.33	347.926	0:03:59	45.00	NA		
PZP	OPD	373.36	19.158	1:12:21	42.00			
Quinalphos	OPD	298.30	21.156	1:05:31	15.45			
Schradan	OPD	286.25	64.786	0:21:24	41.00	0.928	14:56:14	73.8

<table>
<tr><th colspan="3"></th><th colspan="3">Data based on MS</th><th colspan="3">Data based on NMR</th></tr>
<tr><th>OP</th><th>Decay Type</th><th>MW</th><th>Cl_{int}</th><th>Half-life (hour)</th><th>% Remaining</th><th>Cl_{int}</th><th>Half-life (hour)</th><th>% Remaining</th></tr>
<tr><td>TAP</td><td>OPD</td><td>313.31</td><td>5.829</td><td>3:57:47</td><td>52.00</td><td colspan="3">NA</td></tr>
<tr><td>TCP-Fast</td><td rowspan="2">BPD</td><td rowspan="2">257.44</td><td rowspan="2">0.001</td><td rowspan="2">18:01:23</td><td rowspan="2">85.00</td><td>2.506</td><td>6:54:49</td><td>50</td></tr>
<tr><td>TCP-Slow</td><td>0.002</td><td>13:35:34</td><td>13.7</td></tr>
<tr><td>TCVP</td><td>OPD</td><td>365.95</td><td>71.225</td><td>0:19:28</td><td>60.00</td><td colspan="3" rowspan="2">NA</td></tr>
<tr><td>Temephos</td><td>OPD</td><td>466.50</td><td>126.593</td><td>0:10:57</td><td>11.00</td></tr>
<tr><td>Tribufos-Fast</td><td rowspan="2">BPD</td><td rowspan="2">314.50</td><td>219.353</td><td>0:06:19</td><td>10.00</td><td colspan="3" rowspan="2">NA</td></tr>
<tr><td>Tribufos-Slow</td><td>71.265</td><td>0:19:27</td><td>0.14</td></tr>
</table>

^{31}P NMR spectra for formothion and dimethoate (5mM) in the presence of microsomes

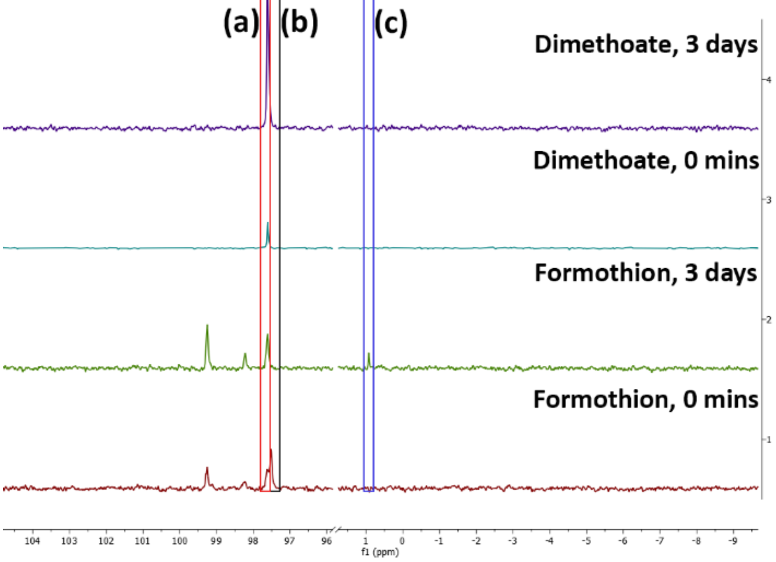

Figure S1: Stacked ^{31}P NMR spectra (in D_2O) of formothion and dimethoate at time points 0 and 3 days: (a) dimethoate δ_P 97.61 ppm present in formothion ^{31}P NMR spectra; (b) formothion δ_P 97.51 ppm present in HLM incubation but absent in dimethoate HLM incubation; (c) minor peak, at δ_P 0.92 ppm, observed in formothion HLM incubation after 3 days.

^{31}P NMR spectra of malathion (5mM) in the presence of microsomes

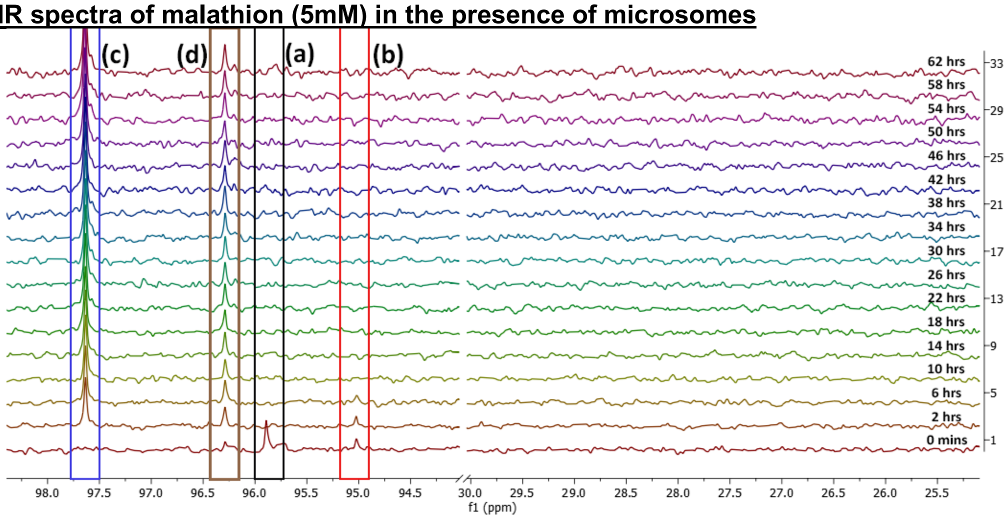

Figure S2: Stacked ^{31}P-NMR spectra of malathion (**4**, D_2O) in the presence of HLMs over a period of days: (a) malathion δ_P 95.89 ppm; (b) major metabolite of **4** δ_P 95.02 ppm that further metabolizes to give metabolites **c** and **d**; (c) other metabolites from **4** and **b** δ_P 97.63 ppm (c) and 96.28 ppm (d).

Commonly observed metabolites for different OPs

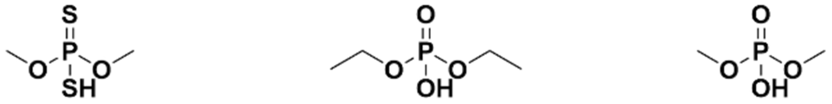

O,O-dimethyl dithiphosphate   Diethyl phosphonic acid   Dimethyl phosphonic acid

Figure S3: Structures of the commonly observed metabolites across different OPs based on their ^{31}P-NMR chemical shifts.

Linear calibration curves of representative OPs for low-dose regimen

Table S3: Calibration curves of representative OPs (At low-dose regimen, MS).

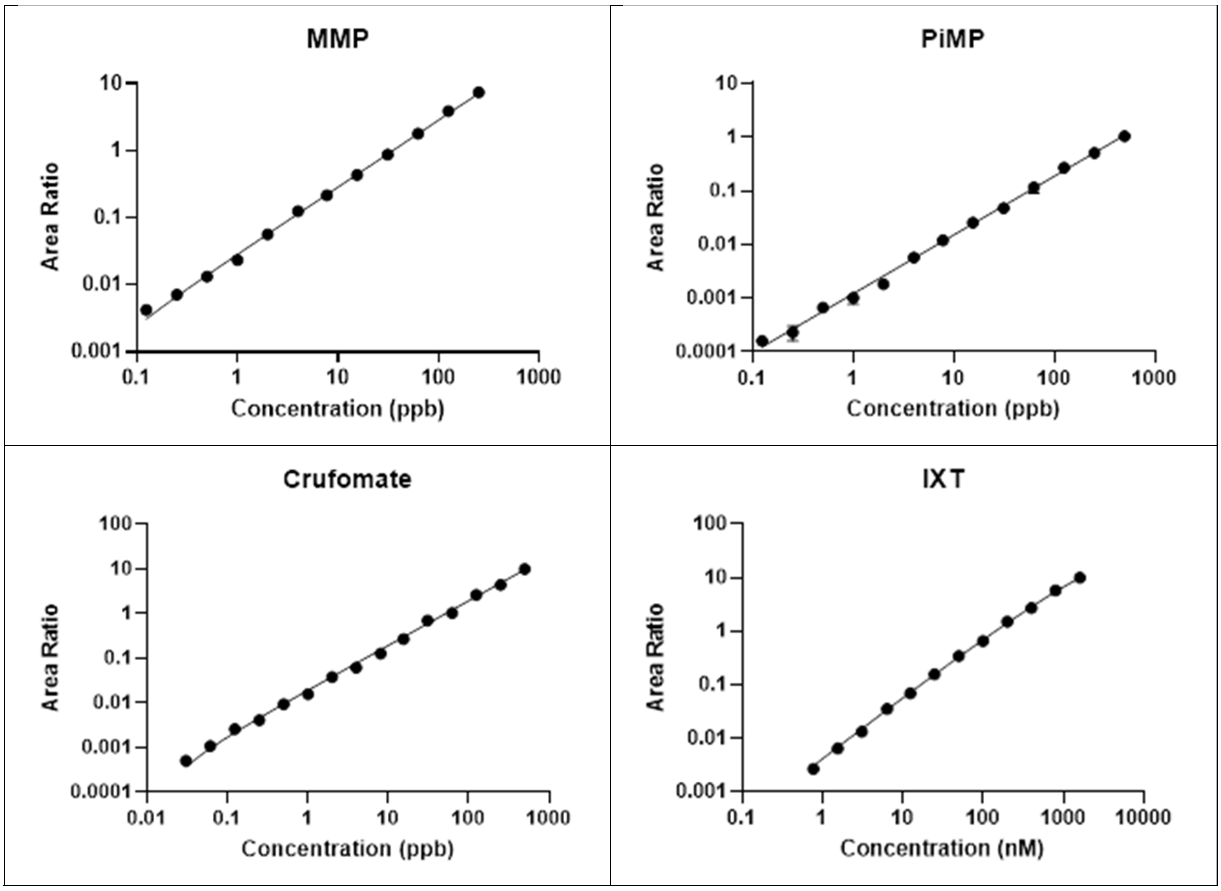

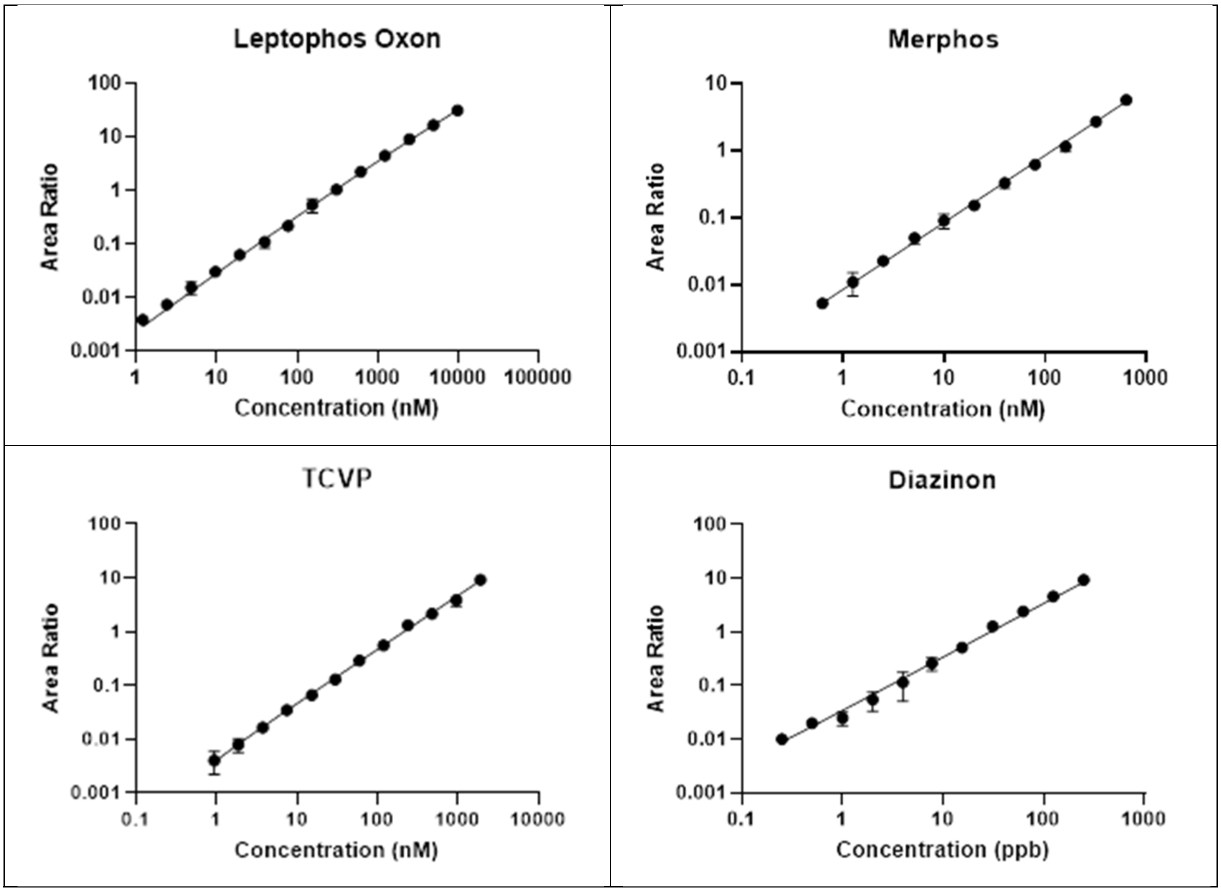

Leptophos Oxon
Area Ratio
Concentration (nM)
Merphos
Area Ratio
Concentration (nM)
TCVP
Area Ratio
Concentration (nM)
Diazinon
Area Ratio
Concentration (ppb)

<u>**Decay curves for OPs at low-(MS) and high-dose (NMR) regimens**</u>

Table S4: Clearance rate graphs of representative OPs.

At low dose regimen (MS)	At high dose regimen (NMR)
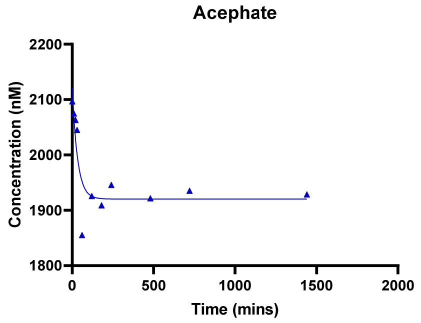	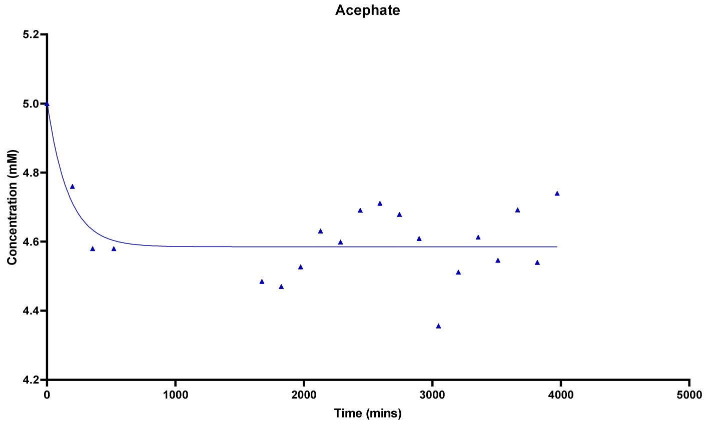
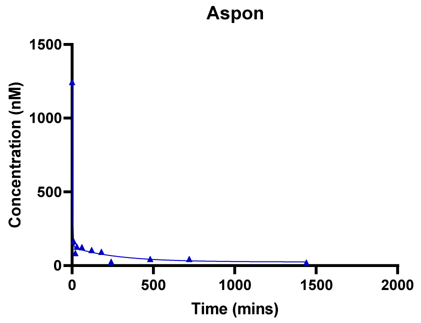	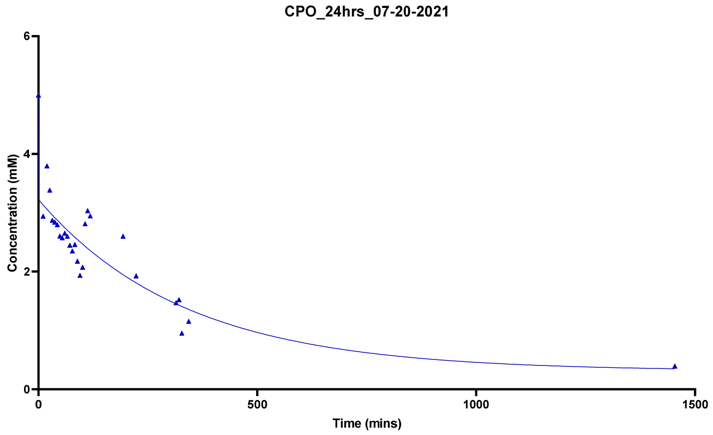
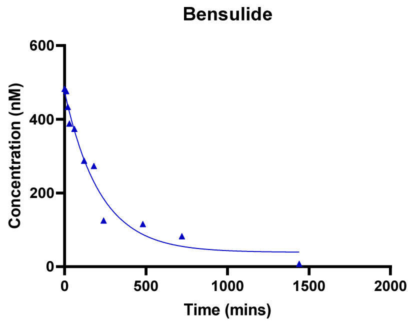	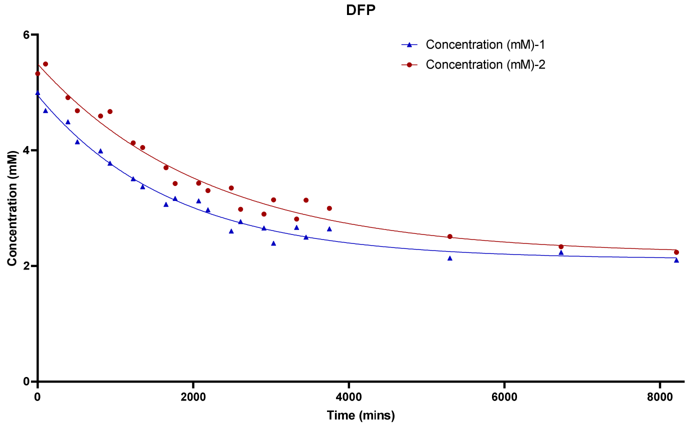

<table>
<tr><th>At low dose regimen (MS)</th><th>At high dose regimen (NMR)</th></tr>
<tr><td>
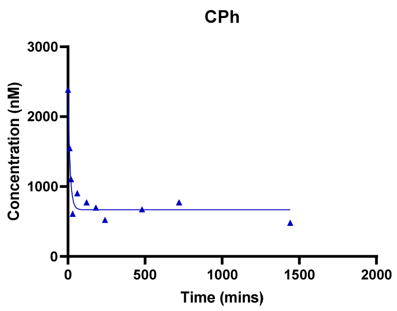

</td><td>
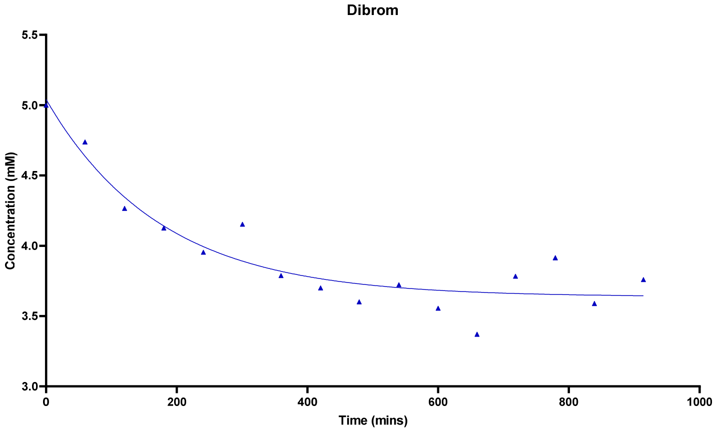

</td></tr>
<tr><td>
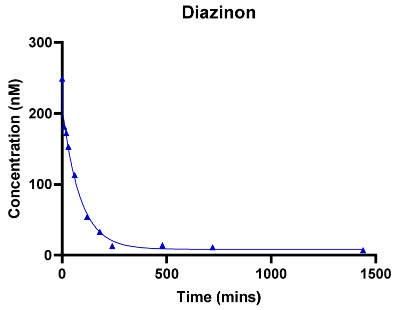

</td><td>
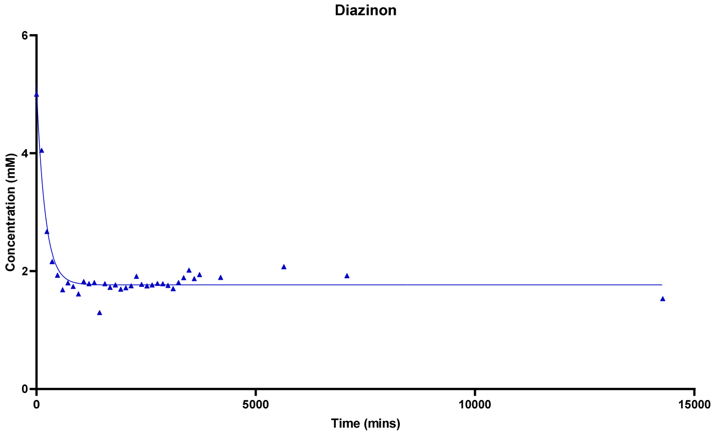

</td></tr>
<tr><td>
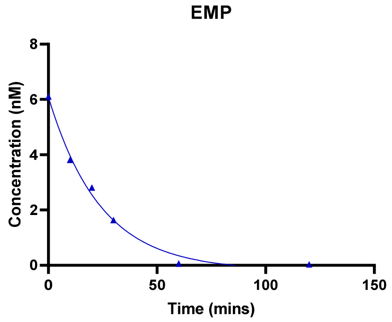

</td><td>
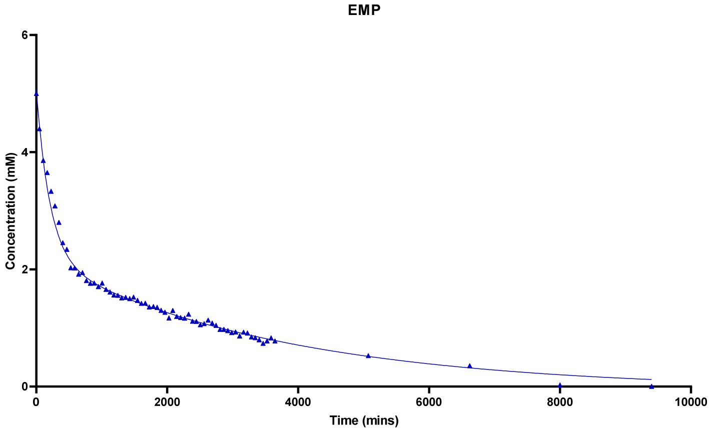

</td></tr>
</table>

At low dose regimen (MS)	At high dose regimen (NMR)
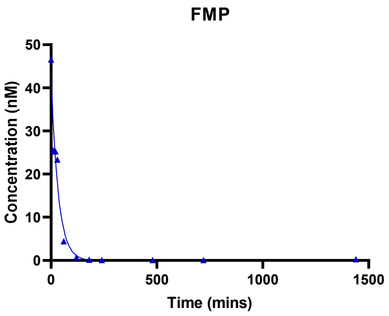	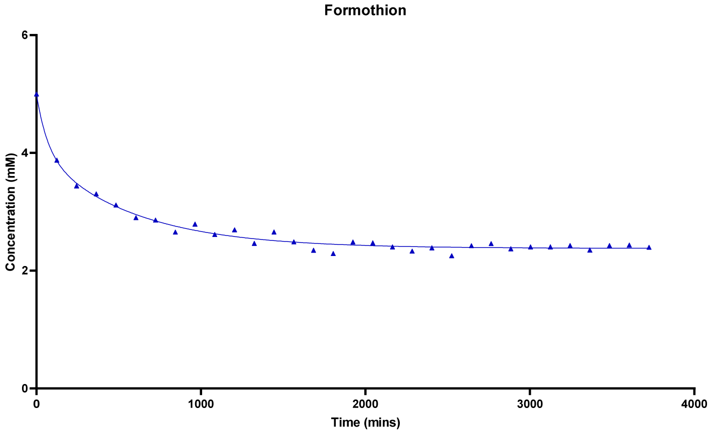
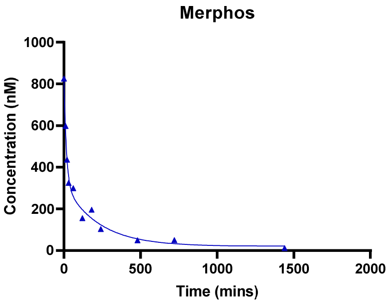	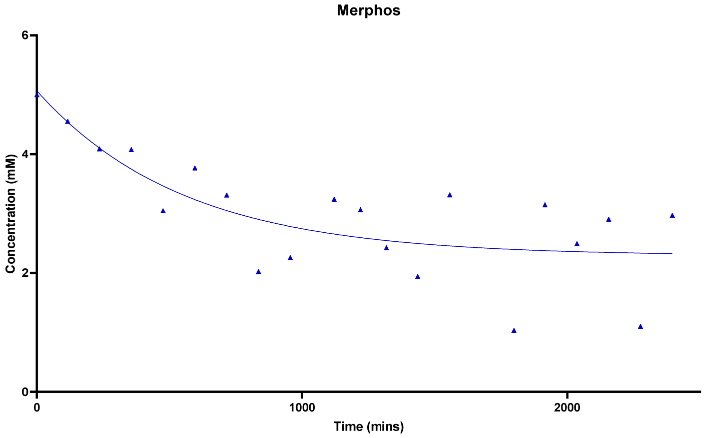
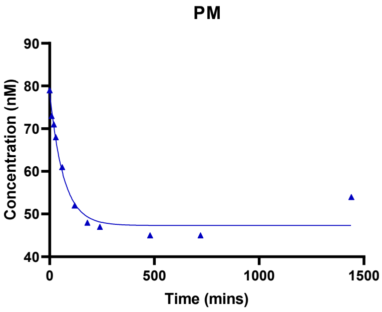	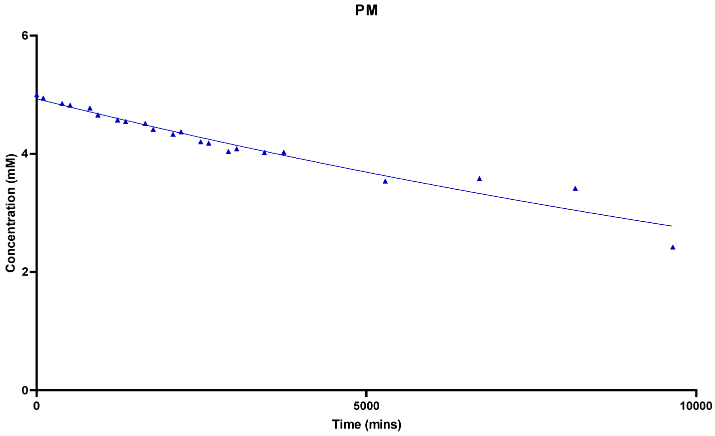

At low dose regimen (MS)	At high dose regimen (NMR)
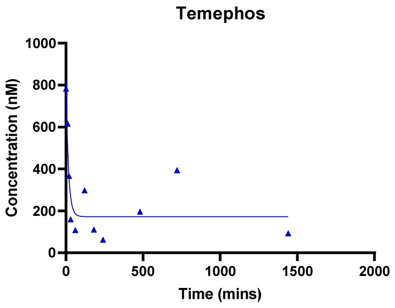	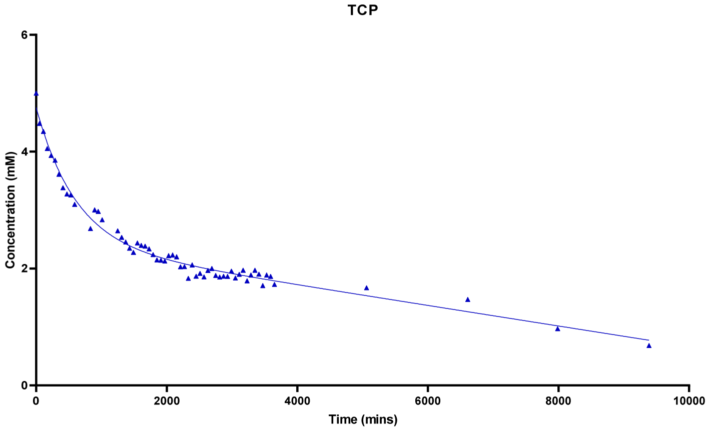
